# Impact of headache on physical activity levels and dynamic balance among university students

**DOI:** 10.3934/Neuroscience.2025022

**Published:** 2025-09-25

**Authors:** Kiruthika Selvakumar, Lee Wan Fei, Deepak Thazhakkattu Vasu, Nandakumari P Velayan Sabapathy

**Affiliations:** Department of Physiotherapy, M Kandiah Faculty of Medical and Health Sciences, Universiti Tunku Abdul Rahman, Selangor, Malaysia

**Keywords:** headache impact, migraine symptoms, dual-task balance, university students, non-pharmacological interventions

## Abstract

**Background and Objective:**

Headache disorders are ranked among the top 10 causes of disability worldwide and are notably prevalent within university student populations. This study aimed to (a) assess the prevalence of headaches and their impact among university students, (b) examine the correlation between headaches and dynamic balance, and (c) investigate the correlation between headaches and levels of physical activity.

**Methods:**

The cross-sectional study was conducted at Universiti Tunku Abdul Rahman and involved 471 participants by using convenience sampling. A digital screening questionnaire was used to obtain demographic data and screen the participants. The Headache Impact Test (HIT-6) and the self-administered short International Physical Activity Questionnaire (IPAQ) Long Last 7 Days were conducted by eligible participants. Timed Up and Go (TUG) and Timed Up and Go Dual Task (TUG-DT; Cognitive and Manual) were assessed to measure dynamic balance.

**Results:**

A 96.9% response rate was obtained in the study. The prevalence of headaches among university students was 44% (n = 208). Spearman correlation analysis indicated very weak to weak correlations that are not statistically significant between headache severity and physical activity levels (r = 0.064, p > 0.05), or with single-task dynamic balance (r = 0.118, p > 0.05) and manual dual-task balance (r = 0.183, p > 0.05). However, a significant positive correlation was observed between headache severity and cognitive dual-task balance performance (r = 0.292, p < 0.05), indicating that greater headache impact is associated with poorer balance under cognitive dual-task conditions.

**Conclusions:**

Headache prevalence among university students was 44%, higher in females and younger individuals. While headache severity showed no significant correlation with physical activity or most dynamic balance measures, it was significantly associated with impaired balance under cognitive dual-task conditions, indicating a potential impact on motor–cognitive integration.

## 1. Introduction

Headaches are among the most common neurological disorders worldwide and significantly impact individuals' quality of life and productivity. They are characterized as pain located in specific regions of the head, such as the frontal, temporal, or occipital lobes, or may be generalized across the entire cranial region [1]. The International Classification of Headache Disorders categorizes headaches into two broad types: primary and secondary. Primary headaches, which include migraine, tension-type headache (TTH), and cluster headache (CH), occur independently and are not caused by other underlying health conditions, whereas secondary headaches are symptomatic of external causes such as infections, trauma, or structural abnormalities [2]. Among these, primary headaches are the most disabling and prevalent, with migraine, CH, and TTH recognized as leading causes of disability globally [2]. In Malaysia, epidemiological data suggest that episodic tension-type headaches affect approximately 25.7% of the population, while chronic forms account for about 1.5% annually [3]. University students are particularly vulnerable to headache disorders due to various stressors such as academic workload, sleep disturbances, prolonged screen time, irregular eating habits, and limited physical activity. One study found that 23.8% of Malaysian undergraduates experienced headaches, with TTH being the most prevalent (12%), followed by migraine (2.4%) and unclassified headache types (8.9%) [4].

Migraines are typically described as unilateral, pulsating headaches accompanied by photophobia, phonophobia, and gastrointestinal symptoms like nausea and vomiting. One proposed mechanism involves cortical spreading depression (CSD), which activates meningeal nociceptors and triggers the trigeminovascular system, leading to neurogenic inflammation and sensitization of dural nociceptors [2],[5]. CH, on the other hand, manifests as severe unilateral pain accompanied by autonomic symptoms such as lacrimation, nasal congestion, and conjunctival injection. These symptoms are mediated by parasympathetic nerve fibers, particularly those associated with the sphenopalatine ganglion and the trigeminal-autonomic reflex arc. During CH attacks, the release of calcitonin gene-related peptide (CGRP) by the Gasserian ganglion plays a critical role in vasodilation and nociceptive sensitization [2]. Tension-type headaches are typically described as a tightening or pressure-like sensation around the head, often likened to wearing a tight band. Psychological stress and fatigue are common triggers, and the underlying mechanisms include nociceptive input from pericranial musculature and heightened central nervous system sensitivity [5],[6].

Despite being largely non-fatal, primary headaches have significant repercussions on the daily functioning and overall well-being of affected individuals, particularly university students. Frequent headache episodes can result in class absenteeism, reduced academic performance, decreased concentration, and increased levels of anxiety [7],[8]. Students often avoid physical activity for fear of triggering headaches, which can contribute to sedentary behavior and decline in physical health [8]. Moreover, reliance on analgesics for acute symptom relief is common, yet chronic or excessive use may lead to medication overuse headache (MOH), a condition where analgesics exacerbate rather than alleviate headache symptoms [4],[9]. MOH, also known as rebound headache, occurs due to the brain's reduced responsiveness to medication following prolonged use, resulting in worsened headache intensity and frequency when medication is withdrawn.

Beyond the cognitive and emotional impacts, recent literature points to a link between primary headaches and impaired dynamic balance and postural control. Postural stability relies on the integration of visual, vestibular, and proprioceptive systems [10]. In patients with TTH and migraine, balance dysfunction is increasingly reported and is believed to result from altered cervical proprioceptive input and dysfunction in central sensory integration [11]. While several studies have evaluated the psychological and academic burden of headaches among university students, relatively few have investigated the direct effects of headaches on physical outcomes such as balance and physical activity. The World Health Organization (WHO) recommends that adults aged 18–64 years engage in at least 150–300 min of moderate-intensity or 75–150 min of vigorous-intensity aerobic physical activity per week, along with muscle-strengthening exercises at least twice weekly (WHO, 2020). University students, however, often fall below these guidelines due to academic pressures, sedentary screen time, and irregular routines. Research in Malaysia, though growing, remains limited in scope. Although. studies have examined headache prevalence in female college students, comprehensive assessments that integrate both physiological and lifestyle-related consequences of headache disorders in diverse student populations are lacking. This represents a significant gap in the literature, particularly as physical health is a key determinant of academic success and quality of life in young adults.

Given these gaps, the current study seeks to examine the prevalence of primary headaches and their association with physical activity levels and dynamic balance among Malaysian university students. Specifically, the study aims to analyze the impact of primary headaches on balance control, as measured by Timed Up and Go (TUG) and Timed Up and Go Dual Task (TUG-DT) tests, and to investigate the relationship between headache severity, as assessed by the Headache Impact Test (HIT-6), and physical activity levels, as measured by the International Physical Activity Questionnaire (IPAQ). Through these objectives, the study aims to contribute meaningful insights into the broader physical implications of headaches in a university setting and inform targeted interventions aimed at improving student health, academic performance, and overall quality of life.

## 2. Methodology

This study employed a cross-sectional design, in which the presence of headaches among students was the independent variable; dependent variables were the outcome measures of the HIT-6, TUG, TUG-DT, and IPAQ. The research was conducted at Universiti Tunku Abdul Rahman (UTAR) Sungai Long campus and Kampar campus. The data collection period spanned five weeks, from October to November 2024. This study was fully self-funded. The study population consisted of undergraduate students. The sample size was determined using the Krejcie and Morgan table, which yielded an initial estimate of 375 participants. Accounting for an anticipated dropout rate of 20% and applying the formula N = N0/(1–DRP), the final required sample size was calculated to be 471. The study was approved by the Scientific Ethical Review Committee (SERC) of University Tunku Abdul Rahman U/SERC/78–363/2024.

Participants were selected through convenience sampling due to its cost-effectiveness and ease of implementation. Researchers approached students in campus common areas and invited them to participate voluntarily. The inclusion criteria were as follows: UTAR students aged between 18 and 25 years, who had experienced headaches in the past month, and who consented to participate. Exclusion criteria included unwillingness to participate and a history of secondary headaches, including those caused by trauma, vascular disorders, infections, or psychiatric conditions, as defined by the International Classification of Headache Disorders, 3rd edition [12].

Outcome measures included the HIT-6, IPAQ, and two functional mobility tests: TUG and TUG-DT. The HIT-6 is a validated six-item questionnaire measuring the effect of headaches on daily activities such as job, study, house chores, and leisure activities, with higher scores indicating greater impact. Each answer represents a score. The total score will define the general impact of headaches on daily life. The IPAQ assesses self-reported physical activity levels through 7 questions to estimate the total physical activity and time spent sitting, categorizing them into low, moderate, or high based on MET-min/week. The TUG test evaluates a dynamic balance by timing a participant's ability to rise from a chair, walk 3 meters, turn, return, and sit down, with a cutoff of 8.57 seconds. The TUG-DT assesses a dual-task performance using either a cognitive task (e.g., counting backward) or a manual task (e.g., carrying a cup of water), with cutoff values of 10.05 and 10.27 seconds, respectively. These tools collectively provide quantitative data on headache impact, physical activity levels, and dynamic balance.

The study procedure involved both online and physical recruitment. A digital questionnaire was distributed via Google Forms through email, WhatsApp, and Microsoft Teams, and administered in person. The form included (1) a research participant information sheet, (2) a personal data protection notice, (3) a consent acknowledgement, (4) demographic data (e.g., age, gender, year, and program of study), and (5) headache screening questions to confirm eligibility.

Eligible participants were scheduled for a single testing session at a physiotherapy center. After obtaining written consent, participants completed the HIT-6 and IPAQ questionnaires. Following this, dynamic balance was assessed using the TUG and TUG-DT tests. Each participant underwent the physical assessments under standardized instructions and supervision to ensure accuracy. This methodology allowed for efficient data collection and assessment of the relationships between headache impact, physical activity level, and dynamic balance in a university student population.

### 2.1. Statistical analysis

All data were entered, coded, and analyzed using Microsoft Excel (Microsoft Corp., USA) and the IBM Statistical Package for Social Sciences (SPSS, Version 26, IBM Corp., Armonk, NY, USA). Descriptive statistics (frequency, percentage, mean, and standard deviation) were employed to summarize the demographic characteristics of the participants, including age, gender, year of study, level of study, and field of study. Prior to inferential testing, data distribution was examined for normality.

Given that the outcome measures did not meet assumptions of normal distribution, nonparametric analyses were performed. Specifically, the relationship between headache impact and outcome measures, namely the HIT-6, IPAQ, TUG, and TUG-DT, was evaluated using Spearman's rank-order correlation coefficient (Spearman's rho). This approach allowed for the identification of the strength and direction of associations between headache severity and participants' physical activity levels and dynamic balance performance under both single-task and dual-task conditions.

## 3. Results

### 3.1. Characteristics of subjects

A total of 486 participants initially completed the pre-screening questionnaire via Google Forms. However, 14 participants declined to share their personal data, resulting in 472 participants who consented to take part in the study. The demographic and academic characteristics included age, gender, study program, year of study, area of study, headache occurrence in the past month, and headache frequency. The data were summarized using frequency, percentage, mean, and standard deviation in Table 1.

### 3.2. Prevalence of headache

The prevalence of headaches among the university students in this study was found to be 44%. Regarding age distribution, the average age of participants who experienced headaches in the past month was 20.71 years. The highest number of affected participants were aged 19 years (n = 50; 24%), while the lowest were aged 24 and 25, with four participants each (1.9%). Gender distribution showed that females had a higher prevalence (116 participants; 55.8%) compared to males (91 participants; 43.8%), with one participant (0.5%) preferring not to disclose their gender. Out of the 472 participants, 410 were excluded from further analysis due to not meeting the inclusion criteria: 264 participants (55.9%) did not experience headaches in the past month, 42 participants (8.9%) were classified under secondary headaches, and 104 participants (22%) refused to complete the study. Ultimately, 62 participants remained and were assessed for outcome measures.

### 3.3. Normality test

Normality of distribution for the outcome measures was evaluated using the Kolmogorov–Smirnov and Shapiro–Wilk tests. Given that the sample size exceeded 50 participants, the Kolmogorov–Smirnov test results were primarily considered. The analysis indicated that 4 out of the 5 variables had p-values less than 0.05, suggesting that the data were not normally distributed.

### 3.4. Descriptive analysis of outcome measure

The outcome measures assessed among the 62 participants included the HIT-6, IPAQ, and dynamic balance tasks: TUG, TUG-DT Cognitive, and TUG-DT Manual. The results were reported using frequency, percentages, means, and standard deviations in Table 2.

**Table 1.** Demographic data.

**Variables**	**n (%) (N = 472)**	**Mean (SD)**
**Age**		20.29 (1.63)
18	54 (11.4)	
19	122 (25.8)	
20	108 (22.9)	
21	81 (17.2)	
22	59 (12.5)	
23	30 (6.4)	
24	10 (2.1)	
25	8 (1.7)	
**Gender**		
Male	207 (43.9)	
Female	256 (54.2)	
Prefer not to say	9 (1.9)	
**Study program**		
Foundation	59 (12.5)	
Undergraduate	410 (86.9)	
Postgraduate	3 (0.6)	
**Year of study**		
Y1	230 (48.7)	
Y2	126 (26.7)	
Y3	70 (14.8)	
Y4	42 (8.9)	
Y5	4 (0.8)	
**Area of study**		
Accounting, Management, Business, and Finance	184 (39.0)	
Arts, Social Science, and Humanities	79 (16.7)	
Medicine and Health Sciences	83 (17.6)	
Science, Technology, Engineering, and Mathematics	126 (26.7)	
**Headache**		
Yes	208 (44.1)	
No	264 (55.9)	
**Frequency**		
<15 days/month	165 (79.3)	
>15 days/month	43 (20.7)	

Note: n = number of participants, SD = standard deviation.

**Table 2.** Descriptive analysis of outcome measure.

**Variables**	**n (%) (N = 62)**	**Mean (SD)**
Headache Impact Test (HIT-6)		2.60 (1.00)
Little or no impact	8 (12.9)	
Some impact	24 (38.7)	
Substantial impact	15 (24.2)	
Severe impact	15 (24.2)	
IPAQ		2.16 (0.81)
Low	16 (25.8)	
Medium	20 (32.3)	
High	26 (41.9)	
TUG		6.58 (1.03)
TUG-DT Cognitive		8.57 (2.05)
TUG-DT Manual		7.47 (1.12)

Note: n = number of participants, SD = standard deviation, HIT-6 = Headache Impact Test, IPAQ = International Physical Activity Questionnaire, TUG = Timed Up and Go, TUG-DT = Timed Up and Go Dual Task.

### 3.5. Correlation between primary headaches and level of physical activity and dynamic balance

Spearman correlation analysis (Table 3) revealed a non-significant but weak positive correlation between headache severity (HIT-6) and physical activity levels (IPAQ) (r = 0.064, p > 0.05), indicating a negligible relationship. Similarly, a non-significant but weak positive correlation was observed between HIT-6 and single-task dynamic balance performance (TUG) (r = 0.118, p > 0.05), or with manual dual-task balance performance (TUG-DT Manual) (r = 0.183, p > 0.05).

In contrast, a statistically significant positive weak correlation was found between headache severity and cognitive dual-task balance performance (TUG-DT Cognitive) (r = 0.292, p < 0.05). This suggests that individuals reporting higher headache impact demonstrated reduced performance under cognitive dual-task conditions.

**Table 3.** Correlation between primary headaches and level of physical activity and dynamic balance (TUG and TUG-DT Cognitive).

	**Mean (SD)**	**r**	**p-value**
HIT-6	2.60 (1.00)	0.064	0.622
IPAQ	2.16 (0.81)		
HIT-6	2.60 (1.00)	0.118	0.361
TUG	6.58 (1.03)		
HIT-6	2.60 (1.00)	0.292*	0.021
TUG-DT Cognitive	8.57 (2.05)		
HIT-6	2.60 (1.00)	0.183	0.154
TUG-DT Manual	7.47 (1.12)		

Note: SD = standard deviation, N = number of participants, r = correlation coefficient, HIT-6 = Headache Impact Test, IPAQ = International Physical Activity Questionnaire.

**Figure 1.**
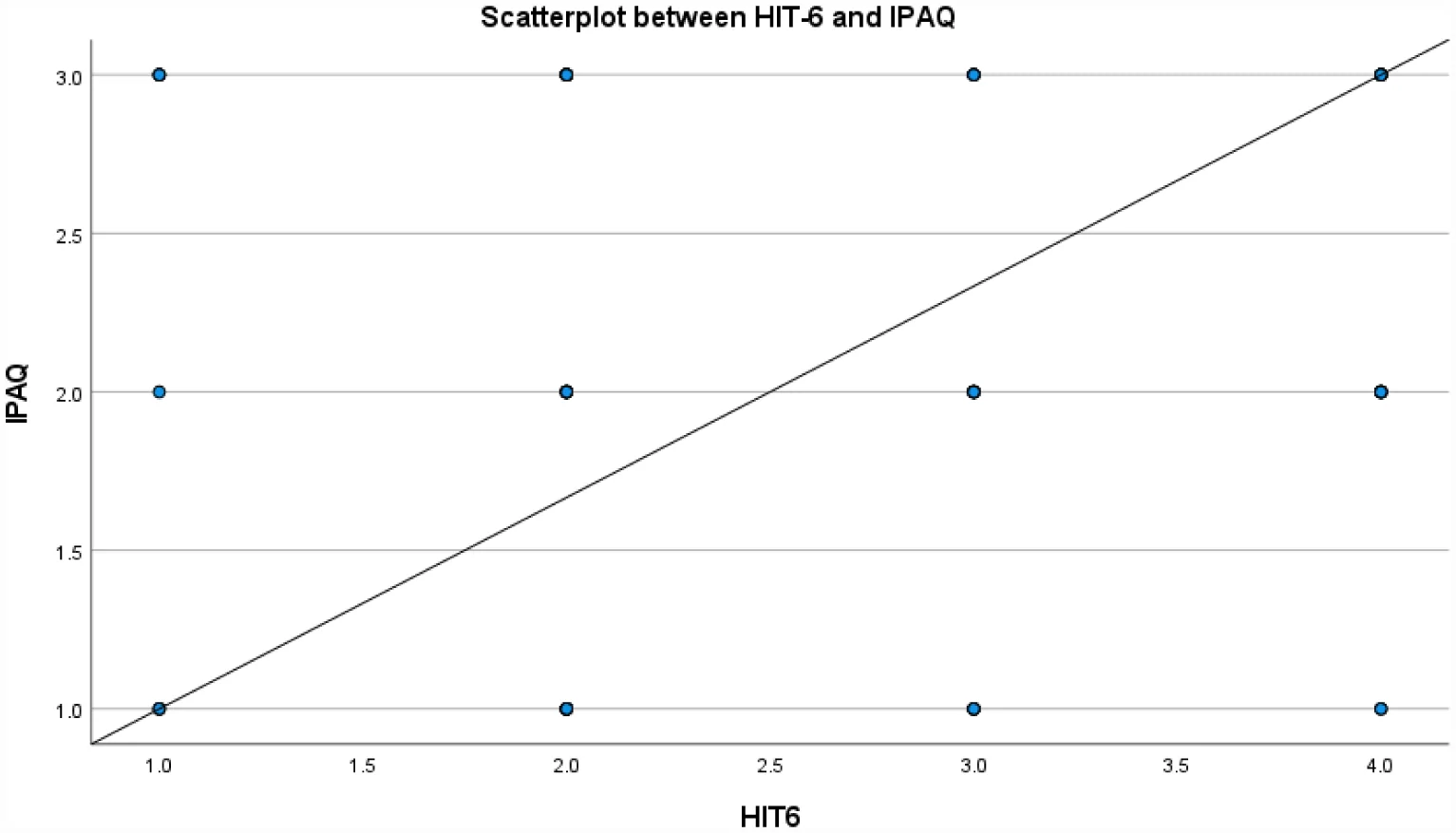
Scatterplot between HIT-6 and IPAQ.

**Figure 2.**
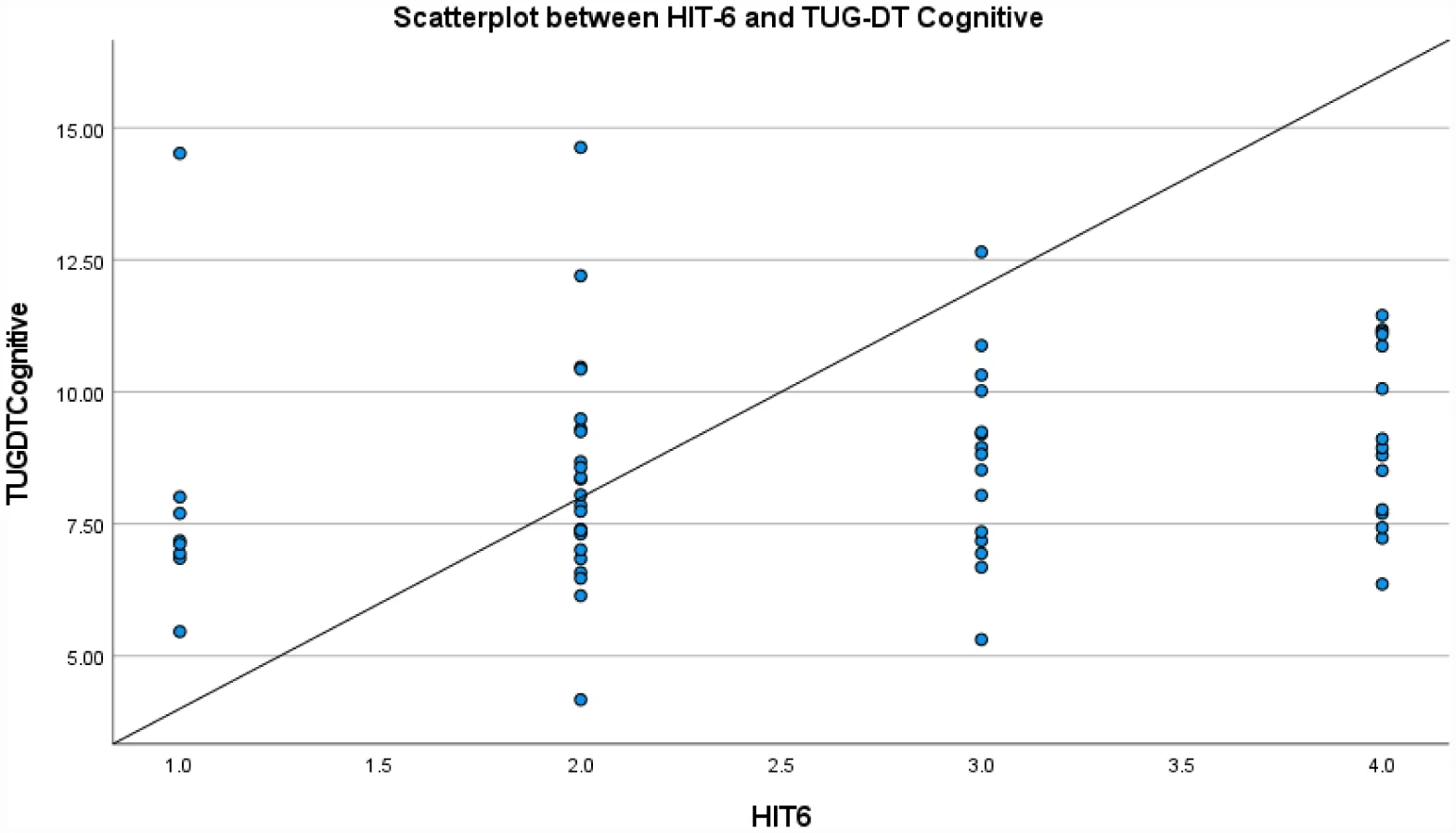
Scatterplot between HIT-6 and TUG-DT Cognitive.

The regression line in Figure 1 shows a slightly positive trend, but the distribution of points does not suggest a strong linear relationship. The regression line of Figure 2 indicates that TUG-DT times increase with higher HIT-6 scores. While there is some spread, the regression line indicates a clearer positive trend. While weak positive trends were observed between headache severity and physical activity, these associations did not reach statistical significance. Given the small analytic sample (N = 62), the study may have been underpowered to detect modest correlations. Therefore, these findings should be interpreted with caution and considered preliminary.

## 4. Discussion

Analysis of correlations between headache severity and outcome measures showed that headaches did not appear to reduce students' physical activity levels, as no significant relationship was found between HIT-6 and IPAQ scores. Similarly, dynamic balance during simple tasks (TUG) was not affected by headache burden. However, when a cognitive task was added (TUG-DT Cognitive), students with more severe headaches performed significantly worse, indicating difficulties in motor-cognitive integration.

The mean age of participants in this study was 20.29 years, aligning closely with similar research among university students. Panigrahi et al. reported a mean age of 21.33 years in undergraduate health profession students [13], while Sanya et al. found a mean age of 20.9 years among undergraduates from three tertiary institutions [4]. In contrast, Almesned et al. observed a higher mean age of 23.15 years among third- and fourth-year medical students, likely reflecting differences in academic cohorts and enrollment patterns across institutions [1]. Regarding gender distribution, females represented 54.2% of participants, consistent with findings by Panigrahi et al. (2020) and Adnyana (2020), who reported female proportions of 65.5% and 59.8%, respectively [13],[14]. However, studies by Sanya et al. (2018) and Almesned et al. (2018) indicated a male predominance, underscoring variability in gender ratios across universities and disciplines [1],[4].

Participants predominantly came from business-related fields such as Accounting, Management, and Finance, with fewer from Arts, Social Sciences, and Humanities. Previous studies have tended to focus on medical and health science students, leaving a gap in knowledge regarding headache prevalence in students from other academic backgrounds [1],[13],[14]. In this study, 44.1% of students reported experiencing headaches within the past month, a lower prevalence than reported by Adnyana (67% within 1–3 months), Panigrahi et al. (73.1% over one year), and Almesned et al. (53.8%) [1],[13],[14]. The variability in prevalence rates may stem from differences in recall periods, sample sizes, academic stress levels, and socio-environmental factors unique to each study population. Notably, 79.3% of participants with recent headaches experienced acute headaches (fewer than 15 days per month), while 20.7% suffered from chronic headaches (more than 15 days per month). This pattern is consistent with previous findings indicating a predominance of acute headaches among university students [4],[14]. However, widespread reliance on over-the-counter analgesics, particularly paracetamol, raises concerns about the potential for medication-overuse headaches, which may exacerbate chronic headache development [9].

A key finding of this study is the identification of a positive, albeit weak, correlation between primary headache occurrence and levels of physical activity. This aligns with previous research by Oliveira et al., Amin et al., and Torres-Ferrus et al., all of whom reported that headaches can negatively impact physical activity participation [8],[15],[16]. Krøll et al. (2018) specifically noted that patients experiencing migraines in conjunction with tension-type headaches and neck pain demonstrate decreased physical activity, higher stress levels, poorer psychological well-being, and overall reduced self-rated health [18]. Amin et al. observed that while exercise might trigger migraine attacks in some individuals, regular physical activity may raise the threshold for migraine triggers and reduce attack frequency, suggesting a complex bidirectional relationship between headaches and exercise behavior [16]. Torres-Ferrus et al. further emphasized that adolescents with headaches often exhibit unhealthy lifestyle habits such as inadequate sleep, poor nutrition, sedentary behavior, and excessive caffeine consumption, factors that are also prevalent in university populations [8]. Consistent with these findings, the current study suggests that students experiencing headaches may limit their physical activity to avoid symptom exacerbation, potentially contributing to reduced fitness and poorer health outcomes. The acceptance of the alternative hypothesis regarding a positive weak correlation, despite the limited sample size, supports the notion that headaches and physical activity are interrelated in this population.

Additionally, this study found a positive weak correlation between primary headaches and dynamic balance, as assessed by the TUG and TUG-DT tests. These results corroborate earlier studies by Carvalho et al. and Dumanlidağ et al., who documented impaired dynamic balance in migraine sufferers [11],[19]. Carvalho et al. reported altered performance in tandem walk and gait tests among patients with migraines, while Dumanlidağ et al. found that individuals with chronic migraines exhibited poorer balance compared to those with episodic migraines. The relationship between primary headaches and balance dysfunction has been explored extensively, with a study conducted in 2005 showing that over half of headache patients present with stabilometric abnormalities [20]. In particular, tension-type headache patients display increased postural sway linked to altered proprioceptive inputs from the craniocervical region. Migraineurs similarly show vestibulospinal system dysfunction manifesting as increased postural sway [20]. So et al. observed that chronic headache patients tend to rely more on vestibular cues and less on visual input for postural control, reflecting a re-weighting of sensory information [21]. Maintaining balance requires the integration of visual, vestibular, and proprioceptive systems; disruptions in these sensory inputs may contribute to both headache pathophysiology and balance impairments. Asai et al. further suggested that migraine and tension-type headache sufferers may experience subclinical abnormalities in subjective visual vertical perception, which can manifest as sensations of imbalance [10]. Notably, this study is among the first to utilize the TUG and TUG-DT tests—comprising cognitive and manual dual-task conditions to investigate dynamic balance in university students with headaches. The consistent positive weak correlations across all TUG measures reinforce the presence of subtle functional impairments associated with headache, even in non-clinical student populations. Taken together, these findings highlight the multifaceted impact of headaches on physical function and underscore the importance of addressing balance and physical activity in headache management strategies for young adults.

A major limitation of this study is the small final sample size, despite a large initial recruitment pool. This substantial attrition, combined with convenience sampling, restricts the representativeness of the findings and reduces statistical power to detect small or moderate associations. Consequently, the results should be interpreted as preliminary and may not be generalizable to the broader Malaysian student population. Future studies should employ larger, randomly selected samples across multiple universities to strengthen external validity. Convenience sampling and recruitment from limited locations may have introduced selection bias and constrained participant diversity. Furthermore, language barriers posed challenges for international students unfamiliar with English, potentially limiting their participation and affecting the sample's heterogeneity. To improve future research, larger, more diverse samples using appropriate random or stratified sampling methods are recommended. Incorporating multilingual questionnaires in English, Chinese, and Malay would facilitate broader participation and more accurate data collection across multicultural university settings.

## 5. Conclusions

The prevalence of headaches among university students was 44%, with a higher burden among females and younger students. The study found that headache severity among university students does not significantly relate to overall physical activity levels or to most measures of dynamic balance. However, headache impact is significantly associated with impaired balance during cognitively demanding dual-task conditions, suggesting that headaches may affect complex motor-cognitive integration. This highlights the need for further research into cognitive-motor interactions in individuals with headache disorders.

## Use of AI tools declaration

The authors declare they have not used Artificial Intelligence (AI) tools in the creation of this article.
